# Forcing a Molecule to Switch: Quantifying Mechanical Control at the Atomic Scale

**DOI:** 10.1021/acs.nanolett.6c01515

**Published:** 2026-06-30

**Authors:** A.M. Shashika D. Wijerathna, Markus Zirnheld, Michael L. Hildebrand, Myles Perry, Marjorie Cenese, Yuan Zhang

**Affiliations:** † Department of Physics, 6042 Old Dominion University, Norfolk, Virginia 23529, United States; ‡ Department of Electrical and Computer Engineering, Old Dominion University, Norfolk, Virginia 23529, United States

**Keywords:** conformational molecular switch, scanning tunneling microscopy, qPlus atomic force microscopy, single-molecule force spectroscopy

## Abstract

Mechanically induced conformational switching at the single-molecule level represents a fundamental mechanism for molecular functionality, yet quantitative characterization of the underlying force and energy landscape remains limited. Here, we study individual TBrPP-Co­(II) molecules on Au(111) using qPlus atomic force microscopy. By reconstructing interaction potentials from 3D Δ*f*(*x*,*y*,*z*) data, we determine a threshold force of ∼96 ± 8 pN and a tip-induced switching interaction energy of ∼38 ± 4 meV associate with the conformational transition. The isolated tip-molecule force follows a power law (exponent ∼6), indicating dominance of long-range van der Waals interactions. At closer distances, deviations reveal force-induced deformation preceding the transition. Validation via the inflection point test confirms measurement reliability. These findings show that long-range dispersive interactions can mechanically deform a molecule and facilitate conformational switching through a deformation-assisted pathway, providing a quantitative framework for controlling mechanically driven functionality at the single-molecule scale.

## Graphical abstract

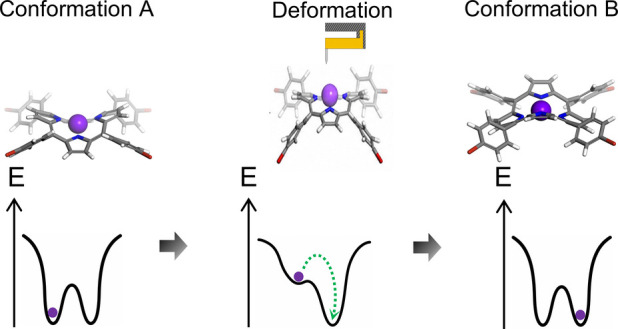

Molecular switches have been intensively investigated over the past decades because they serve as fundamental building blocks for molecular devices, molecular electronics, and nanoscale functional systems.^1–28,42–52^ As envisioned in early molecular electronics concepts, individual molecules are not merely passive conductors but can exhibit intrinsic functionalities that enable device-like behavior at the atomic scale. Among these functionalities, reversible switching between well-defined states represents one of the most appealing and technologically relevant properties. Depending on the intrinsic characteristics of a molecule, switching mechanisms may involve electronic state switching,^1–6^ chemical switching,^7–10^ conformational switching,^11–28^ etc. Among these mechanisms, conformational switching constitutes a particularly important and versatile category. In a conformational switching process, a molecule undergoes geometric reorganization while preserving its bonding topology in response to an external stimulus, and such transitions are often reversible. This molecular-scale ON/OFF behavior can modulate the electronic coupling between the molecule and electrodes, alter transport pathways, or change intermolecular interactions, thereby playing a central role in the development of molecular electronics and device miniaturization.^29–31^ A wide variety of external stimuli have been employed to induce conformational switching, including light irradiation,^11–13^ temperature variation,^14,15^ electric fields,^16–18^ and tunneling electrons.^17–24^ More recently, mechanical force has emerged as an increasingly important stimulus.^25–28^ In densely packed molecular devices or molecular machines, molecules inevitably experience van der Waals interactions and, at shorter distances, chemical forces from neighboring molecules. Mechanical perturbations therefore represent an intrinsic and practically relevant driving force in realistic molecular environments. Understanding how a single molecule responds to mechanical force is thus essential for both fundamental insight and practical device design.

Scanning probe microscopy provides a powerful platform to investigate such processes at the single-molecule level. More specifically, cantilever-based atomic force microscopy and qPlus atomic force microscopy (qPlus AFM) sensors enable controlled application and quantitative measurement of tip-induced forces with subnanometer precision. Although the two AFM techniques have demonstrated remarkable capability in manipulating and characterizing single molecules,^32–41^ examples of mechanically induced conformational switching remain relatively limited. Many reported cases involve lifting a molecule from the substrate to form a tip-molecule-substrate junction, followed by compression or stretching to alter its conformation.^42–48^ Other studies include rotating a molecular subunit^49–51^ and inducing conformational changes via modification of adsorption sites.^52,53^ In contrast, in this work we demonstrate mechanically induced conformational switching of an entire molecule while it remains anchored on the same adsorption site. Using a qPlus AFM sensor, we directly apply controlled mechanical force to the molecule as a whole unit and quantitatively determine the corresponding switching force and interaction energy. This approach provides a direct and quantitative framework for understanding mechanically driven molecular switching at the single-molecule level.

In this study, we investigate TBrPP-Co­(II) [5,10,15,20-tetrakis­(4-bromophenyl)-porphyrin-cobalt], a metalloporphyrin composed of a tetrapyrrole macrocycle coordinating a central cobalt ion and four para-brominated phenyl substituents at the meso positions (Figure 1a). Upon adsorption on Au(111), the molecule adopts a saddle-shaped conformation, in which two opposing pyrrolic units bend upward while the remaining two tilt downward. This distortion arises from a balance between intramolecular steric strain and dispersive interactions with the substrate. The phenyl substituents adjust their dihedral angles to maximize van der Waals coupling with the surface, and this adjustment is directly coupled to bending of the macrocycle.^53–57^ In STM images, the two upward tilted pyrrolic units appear as bright lobes, reflecting their enhanced apparent height (Figure 1b). The brominated phenyl groups provide additional adsorption stability through enhanced dispersive interactions with Au(111), effectively anchoring the molecule at four peripheral contact points. As a result, the adsorbed TBrPP-Co­(II) behaves as a mechanically confined yet elastically deformable nanoscale object: the macrocycle can undergo reversible structural distortion under applied load, while the phenyl “legs” stabilize the adsorption geometry. This balance between flexibility and confinement makes TBrPP-Co­(II) a suitable platform for probing mechanically induced conformational switching at the single-molecule level. When the applied force exceeds a critical threshold, additional mechanical responses such as rotational reorientation or lateral displacement can occur, and these events are occasionally observed experimentally. In most cases, rotational motion follows the conformational switch, while further increases in applied force ultimately leads to lateral translation. However, such events are excluded from the present analysis. Here, we focus exclusively on switching processes in which the molecule remains at the same adsorption site and undergoes only a conformational transition.

**Figure 1:**
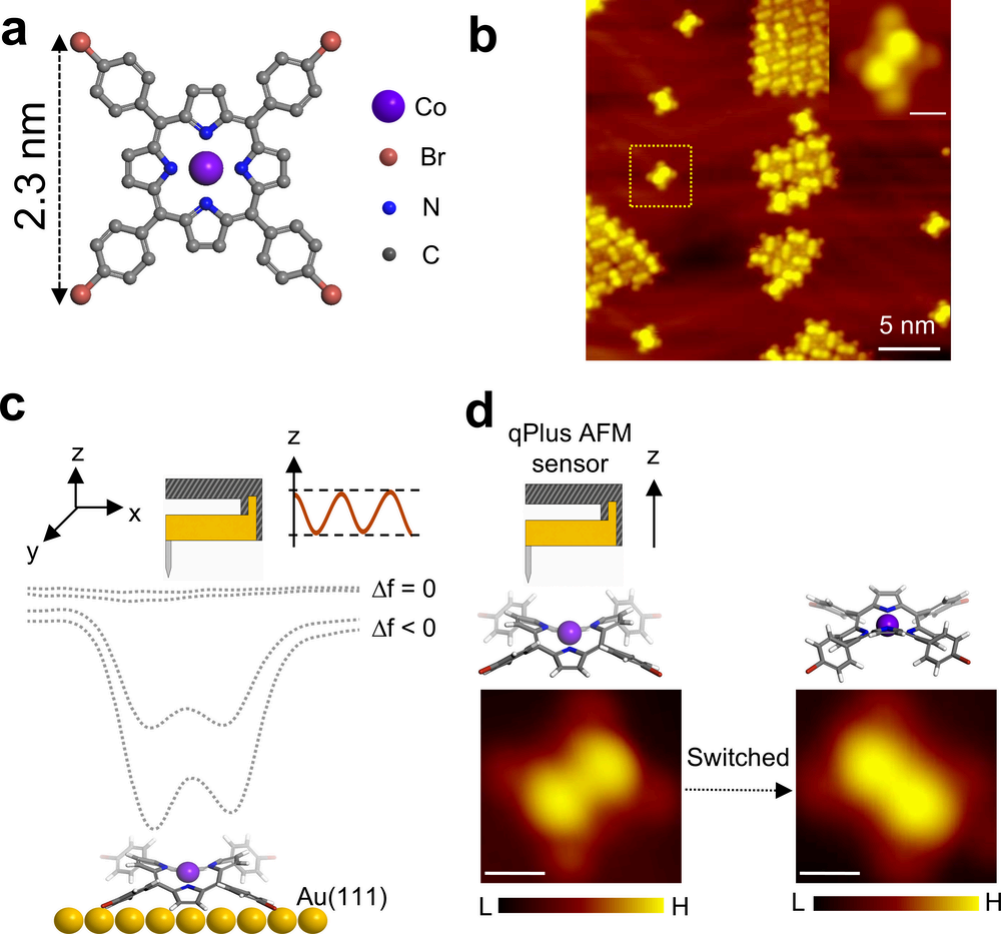
(a) Chemical structure of TBrPP-Co­(II) (hydrogen atoms omitted for clarity). (b) STM image of self-assembled molecular islands and isolated molecules on Au(111); inset shows a magnified view of the molecule indicated by the dashed square. The two bright lobes correspond to the upward-tilted pyrrolic units. (c) Schematic of Δf measurement on a single molecule using a qPlus AFM sensor; the two minima in the Δf line profile arise from interactions with the upward-tilted pyrrolic units. (d) Schematic illustration of conformational switching induced at the threshold tip height, together with STM images before and after switching. Imaging parameters: I = 100 pA, V = −1 V, *T* = 4.6 K. Scale bar: 1 nm.

A Pt/Ir tip mounted on a quartz tuning fork was employed to induce the conformational switching of the molecule and to quantitatively determine the associated switching force and interaction energy. The tip, together with the tuning fork, was driven to oscillate sinusoidally along the *z*-axis (normal to the surface plane) at its natural resonance frequency *f*_*0*_ = 30015 Hz, with an amplitude of *A* = 50 pm. Starting from a relatively large tip-surface distance, the tip was scanned laterally in the *xy*-plane at constant height above a single molecule while recording the frequency shift Δ*f­(x,y,z)* arising from tip-molecule interactions. At large tip-surface separations, the interaction is negligible and Δ*f* ≈ 0. The tip height was then incrementally reduced by Δ*z* = 0.1 Å, and the measurement was repeated (Figure 1c). As the tip approached the molecule, the increasing interaction caused the resonance frequency to deviate progressively from *f*_*0*_. Upon reaching a critical tip height *z*_*th*_, the force exerted by the tip exceeded the mechanical stability threshold of the molecule. At this point, the applied force became sufficient to trigger a conformational switch of the molecule (Figure 1d). Specifically, the pyrrolic units that were initially tilted downward flipped upward, while the previously upward-tilted units bent downward.

Figure 2 illustrates a typical measurement performed following the procedure described above. A single TBrPP-Co­(II) molecule was first imaged in STM topographic mode (Figure 2a). The STM feedback loop was then disabled, and Δ*f­(x,y,z)* maps were recorded in constant-height AFM mode, where *z* denotes the tip height referenced to the Au(111) surface. As shown in the series of Δ*f­(x,y,z)* maps (Figure 2b), no appreciable frequency shift was detected at large tip heights (*z* > 8.6 Å), indicating negligible tip-molecule interaction. As the tip approached the molecule, the molecular contour gradually became visible in the Δ*f* maps, reflecting the increasing interaction strength. This trend is further illustrated by the Δ*f* line profiles taken along the dashed arrow across the molecule (Figure 2e and 2f), where the W-shaped and V-shaped features become increasingly pronounced as the tip height decreases. The two upward-tilted pyrrolic rings, being closer to the tip and therefore interacting more strongly, appear as two depressions in the Δ*f* maps and correspond to the two dips in the W-shaped profiles. The final map in the series, acquired at *z* = 5.9 Å, marks the threshold height for the conformational switch. Immediately afterward, the molecule switches its conformation, as confirmed by the subsequent Δ*f* map obtained at *z* = 5.8 Å (Figure 2d) and the corresponding STM topographic image (Figure 2c). When scanned below this threshold height, the molecule repeatedly switches between the two conformations. Note that the observed contrast change originates from conformational switching rather than a 90° molecular rotation. Detailed experimental evidence is provided in Section 1 of the Supporting Information. A slight asymmetry is observed in the Δ*f* maps, which becomes more pronounced at smaller tip–sample separations. This asymmetry arises from a minor asymmetry of the tip apex. As a result, the molecular center (marked by the cross in Figure 2b) coincides with the central peak of the W-shaped line profiles (Figure 2e) but does not align with the V-shaped curves minima, and the minima of the V-shaped profiles exhibit a slight rightward shift (Figure 2f).

**Figure 2:**
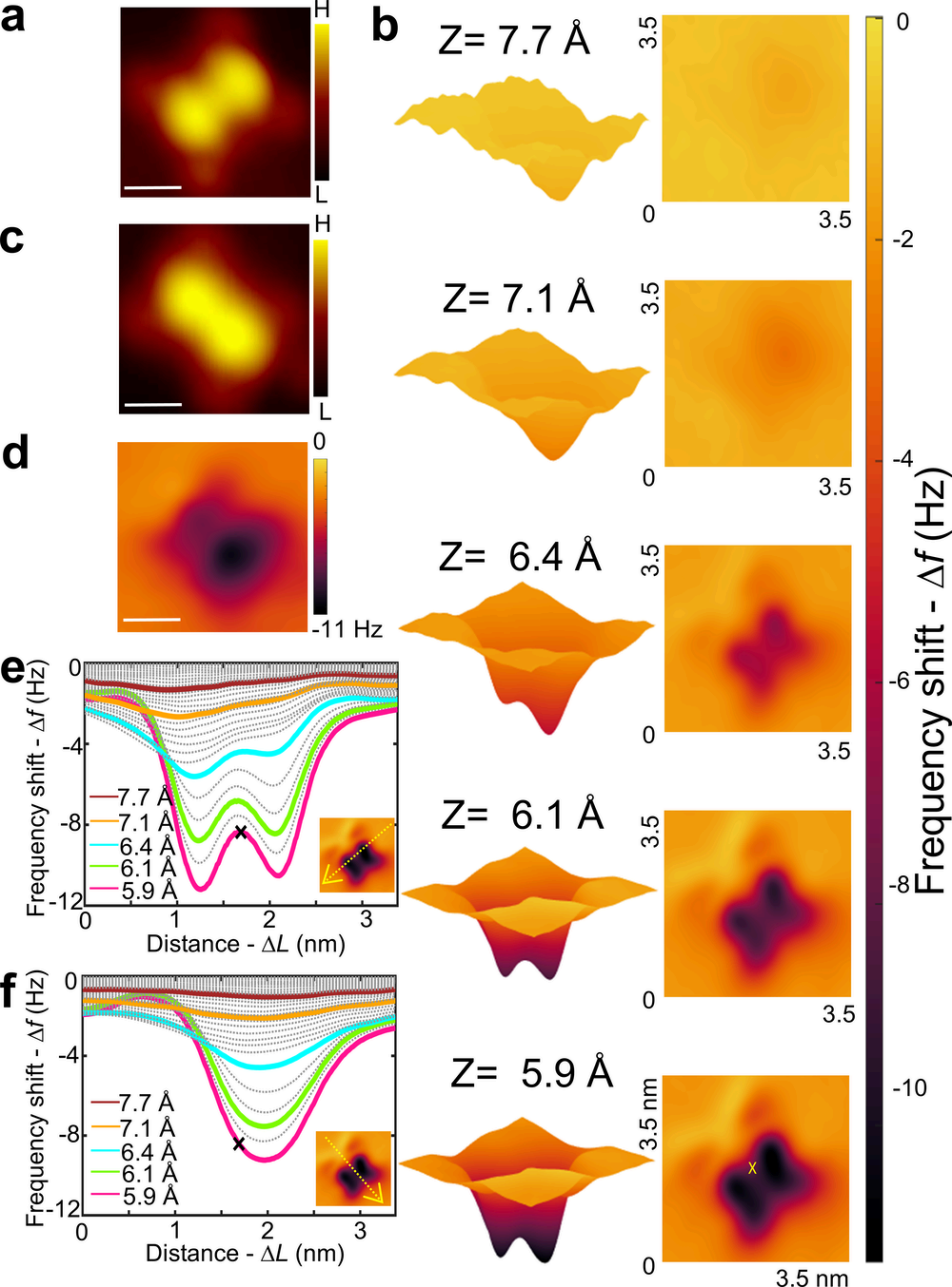
Frequency-shift (Δf) maps acquired at different tip heights over the same TBrPP-Co­(II) molecule. (a,c) STM topographic images of the molecule before and after the conformational switch. (b) Constant-height AFM Δf maps recorded at different tip heights z. The image at z = 5.9 Å corresponds to the last frame before the switching event. The cross (×) marks the molecular center. (d) Δf map obtained immediately after switching at z = 5.8 Å. (e,f) Δf line profiles extracted along the arrow direction in panel (b). Imaging parameters: I = 100 pA; V = −1 V; *T* = 4.6K. Scale bar: 1 nm.

With the series of *Δf* maps recorded at different tip heights above the molecule (Figure 2b), the three-dimensional tip-molecule interaction potential *U­(x,y,z)* was constructed (Figure 3a) using the standard formalism for frequency-modulation atomic force microscopy,^58–60^ as given in the equation below:

1 $$U\left(z\right)=\frac{2k_{0}}{f_{0}}\int_{z}^{\infty }\Delta f\left(z\right)\left[\left(t-z\right)+\sqrt{\frac{A\left(t-z\right)}{16\pi }}+\frac{A^{3/2}}{\sqrt{2\left(t-z\right)}}\right]dt$$

where the sensor was characterized by a stiffness *k*_*0*_ = 1800 N·m^–1^ and a resonance frequency *f*_*0*_ = 30015 Hz.

**Figure 3:**
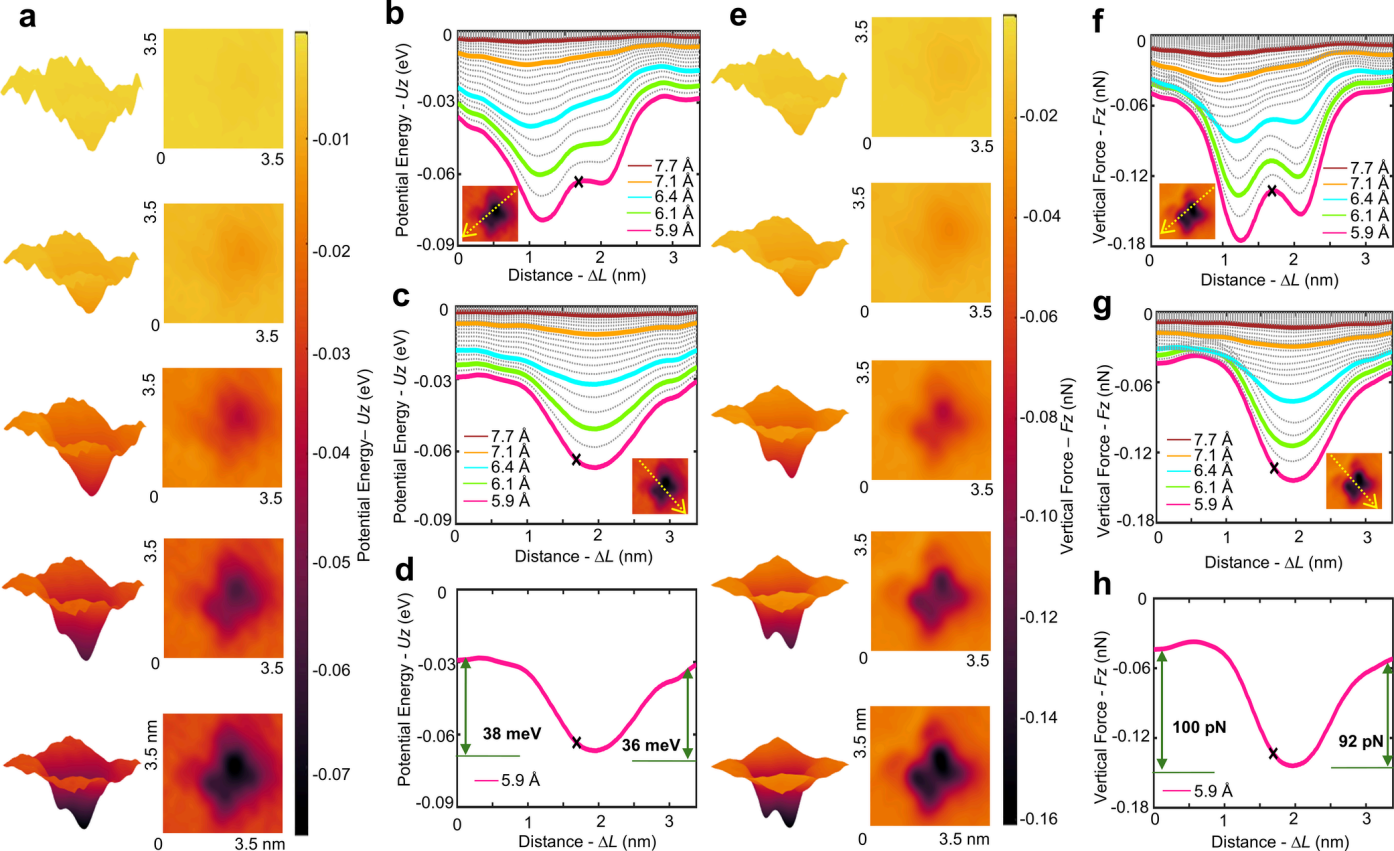
(a) Three-dimensional potential energy map U­(x,y,z) reconstructed from the measured Δf­(x,y,z) data. (b-d) Line profiles of the potential U across the molecule along the direction indicated by the arrow. (e) Three-dimensional force map F_*z*_(x,y,z) obtained by differentiating U­(x,y,z) with respect to the z direction. (f-h) Line profiles of the vertical force F_*z*_ across the molecule along the arrow direction.

Note that acquisition of the complete series of Δ*f* maps requires approximately 10 h. During this time, small lateral thermal drifts could occur even at the measurement temperature of ∼ 4.6 K. To minimize drift-related artifacts, all Δ*f* maps were carefully aligned laterally prior to integration using an image superposition procedure, ensuring consistent reconstruction of the three-dimensional potential map. In addition, the measured *Δf­(x,y,z)* data array is 307 × 307 pixels over an image size of 3.5 × 3.5 nm for the molecule shown here in the *xy* plane, and it contains 28 discrete tip-sample distances along the *z* direction (from *z* = 8.6 Å to 5.9 Å with a decrement *Δz* = 0.1 Å). This step size was selected as a practical balance between resolution and measurement stability: while a finer increment (e.g., Δ*z* = 0.01 Å) would increase the number of *z* points to 280, it would also extend the acquisition time from ∼ 10 h to ∼ 100 h and introduce instability in tip positioning along the *z* direction. Accordingly, a postprocessing approach was employed to increase the effective data density along the *z* axis. Prior to integration to obtain *U­(x,y,z)*, the Δ*f­(z)* curves were interpolated between these 28 points to generate a denser data set along the *z* axis. Specifically, the data set was expanded to 676 points by linear interpolation between adjacent data points, followed by a smooth spline fit^61^ with a smoothing parameter *p* = 0.99 to reduce noise and obtain smooth frequency-shift profiles for subsequent analysis. Previous work by Wang et al. has demonstrated that such postinterpolated Δ*f­(x,y,z)* data sets accurately reproduce the experimentally measured data while maintaining a high level of precision.^35^

Once the potential landscape *U­(x,y,z)* was obtained, the vertical component of the tip-molecule interaction force *F*_*z*_ *(x,y,z)* was then determined (Figure 3e) from the potential by evaluating its derivative with respect to the vertical coordinate *z*. For the single TBrPP-Co­(II) molecule shown here, the vertical force required to induce the conformational switching event was determined to be *F*_*z*_ ≈ 96 pN (Figure 3h), corresponding to an interaction energy of approximately *U* ≈ 37 meV (Figure 3d). Measurements on three different molecules yield an average switching force of approximately *F*_*z*_ ≈ 96 ± 8 pN and a switching interaction energy of *U* ≈ 38 ± 4 meV (see Supporting Information for additional examples). All three molecules were adsorbed at equivalent elbow sites of the Au(111) reconstruction. It should be noted that measurements on different molecules were performed with independently conditioned tips, and thus the tip apex structure may vary between experiments. As a result, the absolute values of the total measured force can differ across data sets. This variation is evident in the Supporting Information, where both the substrate and molecular force magnitudes differ from those in the main text. However, after subtraction of the tip-substrate contribution, the resulting tip-molecule interaction forces show consistent values across different measurements.

It should be noted that the observed transition is not a simple rigid barrier-crossing event, and the measured 38 meV interaction energy should not be interpreted as an intrinsic thermal activation barrier derived from Arrhenius-type switching kinetics. If the intrinsic activation barrier between the two conformational states were comparable to 38 meV, spontaneous thermal switching would be expected to occur more readily at elevated temperatures. However, no spontaneous switching was observed at either 4.2 or 77 K in the absence of tip-induced interaction. Therefore, the measured interaction energy should instead be understood as the tip-induced switching interaction energy associated with the onset of the conformational transition. As will be further discussed below, the force spectroscopy measurements at smaller tip-sample separations suggest that the switching process involves force-induced molecular deformation and a modified switching pathway prior to the transition.

In the next step, we remove the tip-substrate interaction from the total measured force to isolate the force contribution arising solely from the molecule. Following the established ON-OFF subtraction approach commonly used in AFM force spectroscopy studies,^62^ the averaged substrate interaction *F*_*z*_^*tip‑Au*^ *(z)* (Figure 5 inset, red frame), obtained from measurements on the bare substrate away from the molecule, was subtracted from the total interaction *F*_*z*_^*total*^ *(z)* measured above the molecule to obtain the pure tip-molecule interaction: *F*_*z*_^*tip‑molecule*^ *(z)* = *F*_*z*_^*total*^ *(z)* – *F*_*z*_^*tip‑Au*^ *(z)*. Representative extracted tip-molecule interaction curves are shown in Figure 4 (green curves), together with the corresponding total interaction curves (blue curves). After subtraction, the substrate force approaches zero (Figure 4 red frame), confirming effective removal of the substrate contribution.

**Figure 4:**
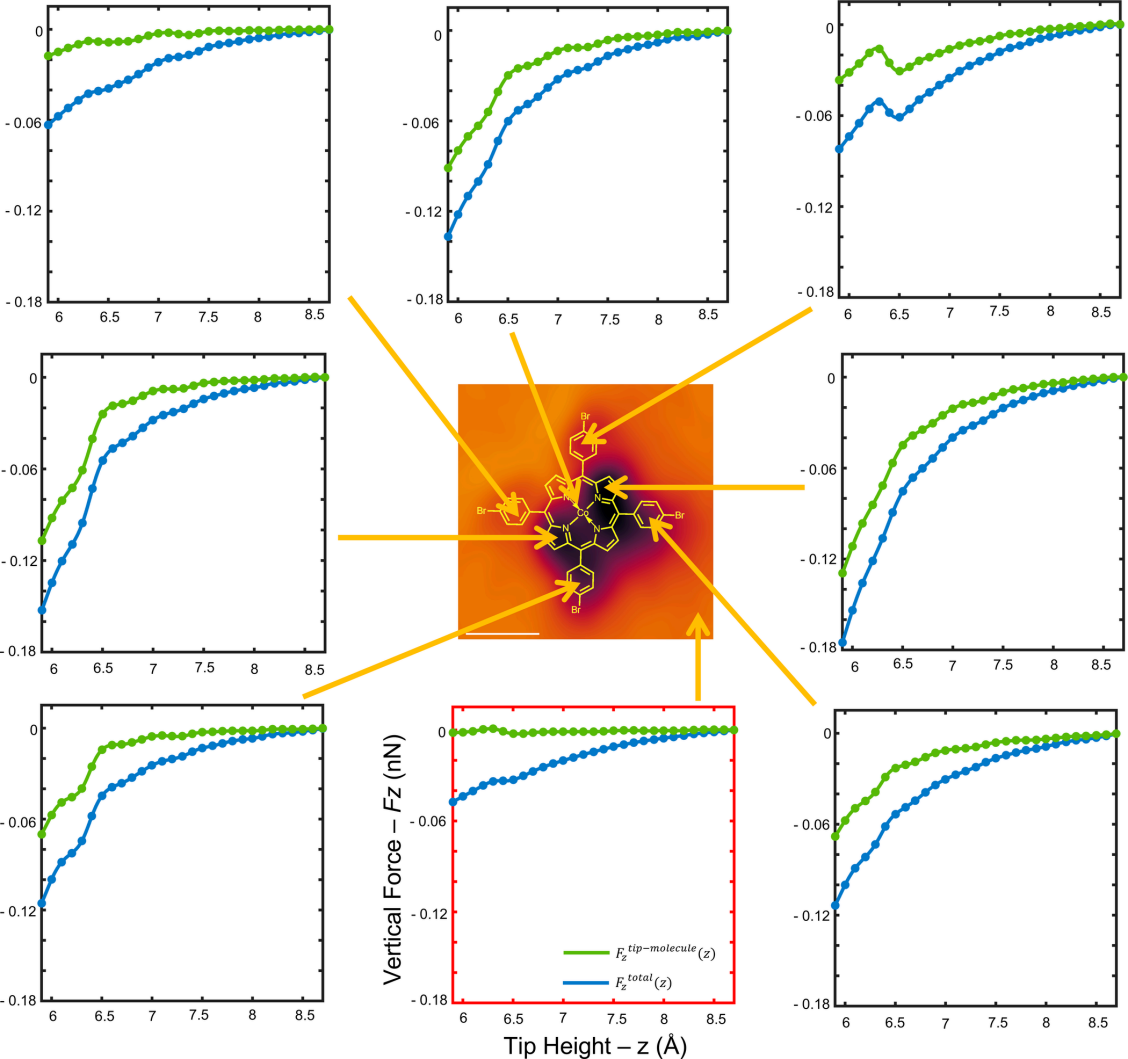
Isolation of tip-molecule force via subtraction of tip-substrate interaction. Total force curves F_*z*_^total^(z) (blue) were acquired at different lateral positions. The substrate contribution, obtained by averaging 10 curves on Au(111) (shown in Figure 5, inset, red frame), is subtracted to yield the net tip-molecule interaction F_*z*_^tip‑molecule^(z) (green). Inset (red frame): the force on Au(111) approaches zero after background removal, confirming effective elimination of the substrate contribution. Molecule structure is overlaid on the force map acquired at the threshold height of z = 5.9 Å. Scale bar: 1 nm.

Following subtraction of the substrate contribution, the extracted tip-molecule force curves were analyzed to characterize the governing interaction regime. The measured forces remain within the attractive regime over the explored separations, indicating that long-range interactions dominate and that the Pauli repulsive regime is not reached.^63^ Under such conditions, fitting with a full Lennard-Jones (LJ) or modified LJ potential is not appropriate, as such models require sampling of both attractive and repulsive branches to uniquely constrain the equilibrium distance and energy minimum. Instead, we employ a power-law representation,

2 $$F\left(z\right)=A{\left(z+d\right)}^{-\left(g+1\right)}+B$$

which captures the asymptotic behavior of long-range interactions. Consistent with the notation used throughout this article, *z* denotes the tip-substrate distance, with the absolute tip height calibrated from averaged Δ*f­(z)* curves measured on the bare Au(111) surface, while *d* represents an effective offset in the tip-sample separation.

Shown in Figure 5, over the molecule, the fitting is restricted to the range *z* = 6.5 ∼ 8.6 Å, where the force curves follow a consistent long-range attractive trend. Within this range, the exponent g ≈ 6 over the molecule indicates a dominant van der Waals interaction with the expected *z* ^*–6*^ dependence.^64^ The fitted offset *d* < 0 reflects the reduced effective tip separation relative to the substrate due to molecular height. Moreover, *d* becomes increasingly negative at protruding molecular features compared to the central region and peripheral legs, consistent with the expected topographic variation in tip-sample distance. In contrast, for the bare Au(111) surface, the entire *z* range can be fitted, as no molecular deformation is involved to distort the force profile. In this case, we obtain *g* ≈ 4.5, consistent with a superposition of van der Waals and longer-range electrostatic interactions, which effectively reduce the decay exponent.^63–65^ These results are in good agreement with established AFM force spectroscopy studies of molecular adsorbates and metallic substrates.

**Figure 5:**
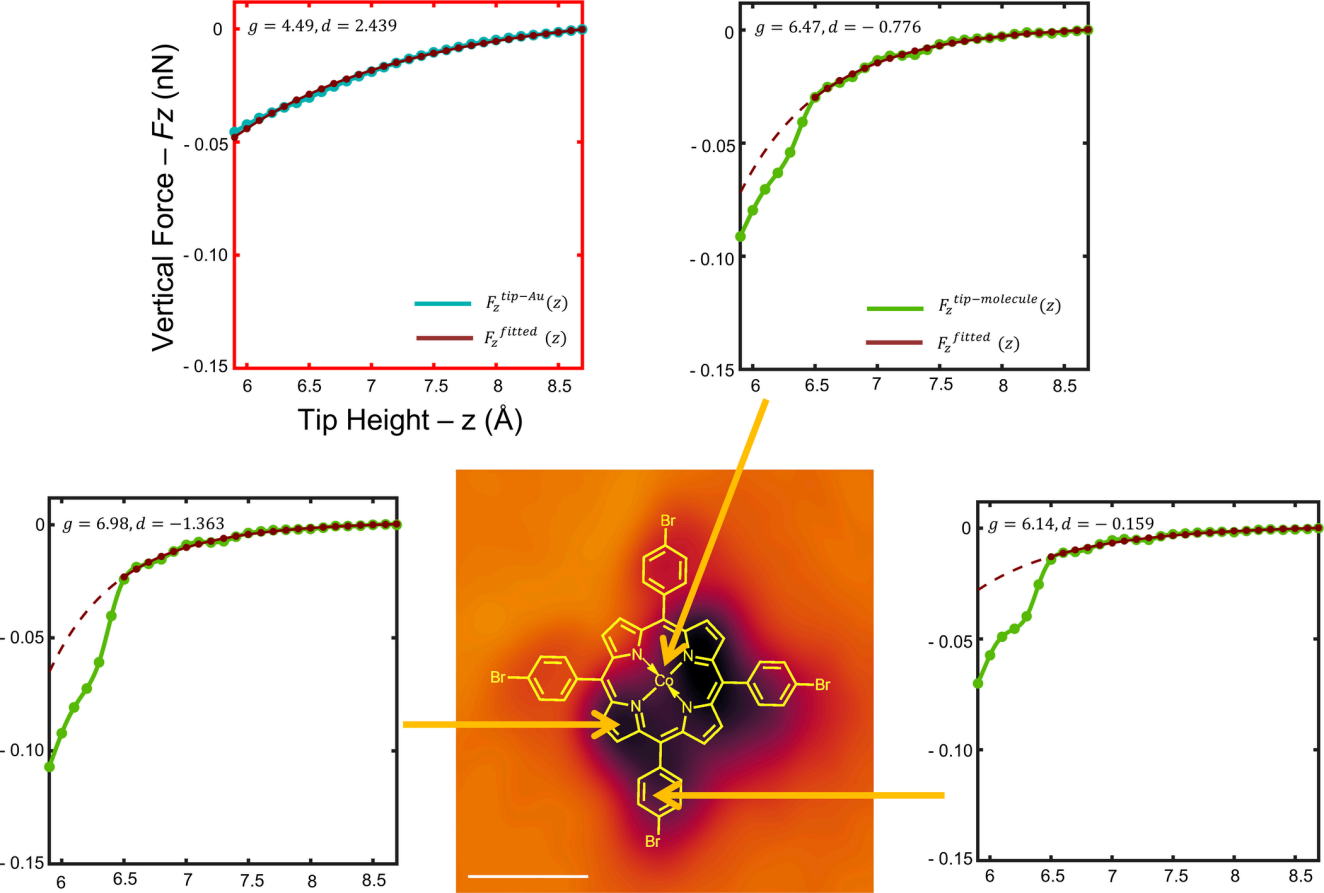
Background-subtracted tip-molecule force curves F_*z*_^tip‑molecule^(z) (green) measured at representative positions on the molecule, together with power-law fits (maroon) over the range z = 6.5–8.6 Å. Inset in red frame shows the averaged tip–substrate force F_*z*_^tip‑Au^(z) used for background subtraction, together with its corresponding fit. Scale bar: 1 nm.

At smaller tip height (*z* < 6.5 Å), clear deviations from the power-law trend are observed (Figure 5). This behavior is also evident in the force curves shown in Figure 4, where a sudden drop occurs around z ≈ 6.5 Å for most lateral positions on the molecule, indicating the onset of a mechanical response as the molecule begins to deform under the tip-induced force prior to a complete conformational switch. In addition, the force curve acquired on one of the molecular legs (Figure 4, top-right) exhibits a distinct bump around z ≈ 6.5 Å, which is attributed to a localized structural rearrangement, specifically, a rotation of the leg followed by relaxation. These features confirm that the molecule is not structurally rigid and responds mechanically to the approaching tip. Although short-range chemical attractive interactions may also contribute to this regime and lead to deviations from the *z* ^*–6*^ ideal behavior, the observed sudden drop and localized bump are more consistently explained by force-induced molecular deformation. Accordingly, only the nonperturbed regime is used for fitting to extract intrinsic interaction parameters.

Further insight can be obtained by comparing the measured force curves (green) with the extrapolated power-law fits. In the deformation regime, the measured force lies below the fitted curve, indicating an effective reduction in the tip-molecule separation due to deformation. This suggests that, although the molecular legs remain anchored to the substrate, the molecular framework is pulled toward the tip, enhancing the interaction before relaxation and the eventual switching. The pronounced discrepancy between the measured and fitted curves reflects the magnitude of this deformation. In contrast, for the additional example presented in the Supporting Information, the deviation from the fitted curve is much less pronounced and the measured force remains close to the fitted behavior up to the transition, indicating that the deformation process is relatively small and short-lived. There, the measured force (green curve) stays above the extrapolated fit in the deformation regime, in contrast to the main-text example where it lies below the fit. This difference reflects the absence of significant upward deformation toward the tip. Instead, the pyrrolic unit transitions downward more directly, leading to an increased tip-molecule separation and thus a reduced attractive interaction prior to switching.

To summarize our force spectroscopy analysis at both large and short tip-sample separations, the long-range tip-molecule interaction is predominantly governed by dispersive van der Waals forces, as supported by the observed power-law behavior in the nonperturbed regime (*z* = 6.5–8.6 Å). However, as the tip approaches below *z* < 6.5 Å, clear deviations from the idealized power-law behavior emerge together with signatures of localized structural response prior to switching. These observations indicate that the molecule is no longer mechanically rigid in the intermediate switching regime. Instead, the tip-induced dispersive interaction modifies the local energy landscape and induces molecular deformation during the approach process. The deformation plays a critical role in facilitating the conformational transition through a deformation-assisted switching pathway rather than a simple rigid barrier-crossing process. As discussed above, the measured 38 meV therefore corresponds to the tip-induced interaction energy associated with the onset of switching, rather than an intrinsic thermal activation barrier between two rigid conformational states. In addition, although different molecules may exhibit different deformation trajectories during the approach process, the extracted threshold force and tip-induced switching interaction energy remain highly consistent across measurements. This observation suggests that molecular deformation in the present system acts as a mechanical trigger that drives the molecule toward a reproducible critical switching condition prior to the conformational transition.

A more complete microscopic understanding of the reshaped energy landscape, deformation trajectory, and detailed switching pathway would require complementary theoretical modeling and simulations. In particular, a meaningful theoretical description would need to simultaneously account for force-induced molecular deformation, the dynamically modified energy landscape, and the dependence of the switching pathway on the local force application geometry. These considerations make the microscopic modeling substantially more complex than a simple static rigid barrier-crossing problem. The present work therefore focuses on experimentally quantifying the threshold force and tip-induced switching interaction energy associated with mechanically induced conformational switching, which provides a quantitative experimental foundation for future theoretical investigations. Importantly, the experimentally observed deviations from the idealized long-range interaction behavior exhibit a strong dependence on tip-molecule distance and spatial tip position. Combined with the dense spatial resolution of the 3D *Δf­(x,y,z)* mapping spectroscopy, these measurements contain rich quantitative information regarding the evolution of the molecular deformation process during tip approach. In principle, such experimentally resolved spatially dependent deviations may provide important quantitative constraints for future theoretical modeling and simulations aimed at reconstructing the detailed deformation pathway and the associated reshaped energy landscape prior to switching.

Unlike many AFM manipulation studies employing functionalized tips that access both long-range interactions and strong short-range chemical attractive and repulsive interaction regimes, the present measurements were performed using a bare metal tip to more directly isolate and investigate the long-range van der Waals interaction regime prior to direct chemical interaction. A key conceptual advance of the present work is the experimental demonstration that long-range dispersive interactions can mechanically deform a molecule and initiate conformational switching through a deformation-assisted pathway. More broadly, this interaction regime may also more closely resemble the initial stages of force coupling in realistic molecular-scale functional devices, where dispersive interactions naturally precede strong chemical attractive and repulsive interactions.

In addition, the present molecular system exhibits an important balance between conformational stability and mechanical flexibility. No spontaneous switching was observed at either 4.2 or 77 K in the absence of external perturbation, indicating that the molecular conformations remain thermally stable. At the same time, the switching process remains mechanically accessible once sufficient deformation is induced by the tip-molecule interaction. This combination of thermal stability and controllable mechanical actuation is particularly attractive for future molecular-scale functional systems and further highlights the importance of considering molecular deformation and force-modified energy landscapes in the design and interpretation of mechanically driven molecular switching processes.

Finally, we evaluated the reliability of the reconstructed force curves using the inflection point test following the formalism introduced by Sader et al.^66^ and experimentally implemented by Huber and Giessibl.^67^ In dynamic AFM, a key criterion for assessing the well-posed condition of the measurement is the behavior of the force near its inflection point, where the curvature (the second derivative of the force) changes sign. If the characteristic length scale of force variation at the inflection point is comparable to or smaller than the tip oscillation amplitude, information is effectively blurred during the measurement, leading to unreliable force reconstruction. To ensure the validity of our results, we employed a small oscillation amplitude *A* = 50 pm and we applied this inflection point test to all reconstructed force curves. The analysis confirms that our measurements satisfy the well-posed condition, with no indication of instability or spurious oscillations in the recovered force. In particular, the smooth, monotonic behavior of the force curves in the analyzed regime indicates that the characteristic length scale of variation exceeds the oscillation amplitude, thereby avoiding the ill-posed regime. Details of this analysis are provided in the Supporting Information. These results validate that the extracted force profiles reliably reflect the underlying tip-sample interaction.

In summary, we have demonstrated mechanically induced conformational switching of a single TBrPP-Co­(II) molecule on Au(111), while maintaining the molecule at a fixed adsorption site. By reconstructing the three-dimensional interaction potential from Δ*f­(x,y,z)* measurements, we quantitatively determined both the switching force and the associated interaction energy, yielding values of approximately 96 pN and 38 meV, respectively. This direct measurement provides a rare quantitative description of force-driven molecular switching at the single-molecule level.

Through systematic separation of tip-molecule and tip-substrate interactions, we reveal that the force-distance behavior follows a power-law dependence with an exponent g ≈ 6 at large tip-sample separations. At smaller tip-sample separations, deviations from this behavior are attributed to force-induced molecular deformation and structural rearrangements preceding the switching event. These observations highlight that the tip-induced dispersive interaction modifies the local energy landscape during the approach process and induces molecular deformation prior to switching. The deformation then facilitates the conformational transition through a deformation-assisted pathway within a modified local energy landscape. Importantly, we validate the reliability of the reconstructed force curves using the inflection point test, confirming that the measurements are well-posed and free from inversion artifacts. This methodological rigor ensures that the extracted force profiles faithfully represent the underlying physical interactions.

Overall, this work establishes a quantitative framework for probing mechanically induced molecular switching and provides new insight into how nanoscale mechanical forces govern functional behavior in single molecules. The approach is broadly applicable to other molecular systems and offers a pathway toward rational design and control of mechanically responsive molecular devices.

## Experimental Method

All experiments were performed using a CreaTec low-temperature (∼5 K) ultrahigh vacuum (UHV) (base pressure ∼ 8 × 10^–11^ mbar) STM/AFM. An Au(111) single-crystal substrate was prepared by repeated cycles of Ar^+^ sputtering (1 × 10^–5^ mbar, 1 keV) followed by annealing to ∼ 675 °C and subsequently cooled to ∼ 5 K in the microscope. TBrPP-Co­(II) molecules (99.9% purity) were thermally deposited onto the clean Au(111) surface from a resistively heated tantalum crucible under UHV conditions. The Pt/Ir tip was chemically etched in CaCl_2_ solution prior to installation and further sharpened in situ by controlled nanoindentation into the Au(111) surface combined with bias voltage pulsing.

## Supplementary Material



## Notes

Distinguishing conformational switching from molecular rotation; Comparison of force measurements across different molecules; inflection point test (PDF)
