# Supplementary material for: Forcing a Molecule to Switch: Quantifying Mechanical Control at the Atomic Scale
Source: Nano Lett. 2026 Jun 30;26(27):8661–70. doi: 10.1021/acs.nanolett.6c01515 (PMC13377593; doi:10.1021/acs.nanolett.6c01515)
Supplement: Supplementary file 1 [file nl6c01515_si_001.pdf]

Supplement information

Forcing a Molecule to Switch: Quantifying Mechanical Control at the Atomic Scale

A.M. Shashika D. Wijerathna[1], Markus Zirnheld[1], Michael L. Hildebrand[1], Myles Perry[2], Marjorie Cenese[2], Yuan Zhang[1]*

AUTHOR ADDRESS

1 Department of Physics, Old Dominion University, Norfolk, Virginia, 23529, United States.

2 Department of Electrical and Computer Engineering, Old Dominion University, Norfolk, Virginia, 23529, United States.

This Supplement information contains the details for:

1. Distinguishing conformational switching from molecular rotation.
2. Comparison of force measurements across different molecules.
3. Inflection point test.

1. Distinguishing conformational switching from molecular rotation

An important question is whether the observed switching events correspond to conformational switching or simply rigid-body molecular rotation on the Au(111) surface. Here, we clarify the experimental distinction between these two processes. Figure S1a shows a large-area STM image containing both isolated molecules and self-assembled molecular islands on Au(111). For isolated molecules, the two upward-tilted pyrrolic units preferentially align along the close-packed $[1\bar{1}0]$ direction of the Au(111) surface, as highlighted by the green guideline. This preferred adsorption orientation remains preserved within the molecular islands. However, intermolecular interactions additionally influence the assembly geometry, resulting in neighboring molecules alternating their orientations by approximately 90°, corresponding to alternating alignment along the $[11\bar{2}]$ directions, as indicated by the blue guidelines. Since isolated molecules preferentially align along the close-packed directions, experimentally observed rigid-body rotational events under tip manipulation always occur through discrete 60° rotational steps rather than 90° rotation. This behavior is demonstrated in Figure S1b–d, which shows representative STM images obtained during sequential manipulation of the same molecule, where the molecular orientation changes through successive 60° rotational steps consistent with the preferred adsorption registry on Au(111). Therefore, the switching events analyzed in the present work can be readily distinguished from rigid-body rotation.

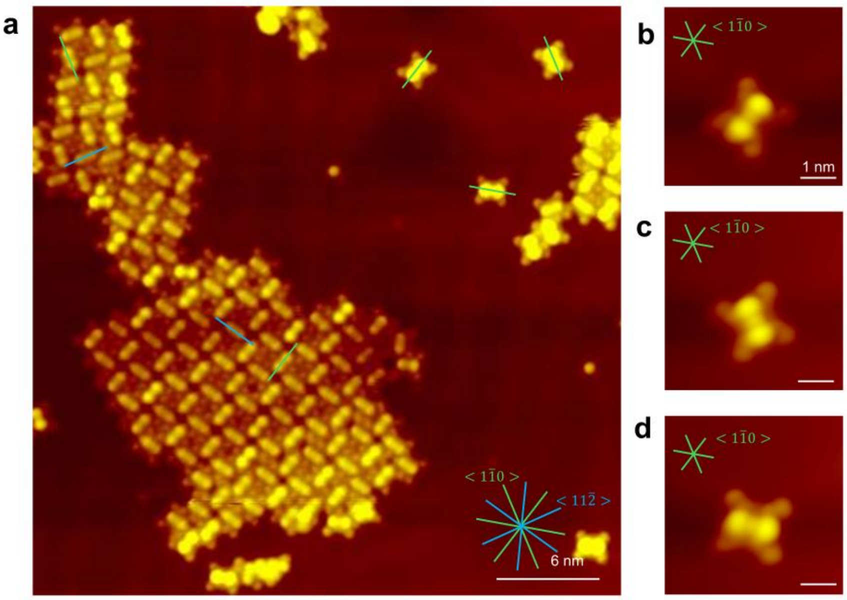

Figure S1. (a) Large-area STM image showing both isolated TBrPP-Co(II) molecules and self-assembled molecular islands on Au(111). Green lines $[1\bar{1}0]$, blue lines $[11\bar{2}]$. (b–d) Representative STM images obtained during sequential tip-induced manipulation of the same molecule.

2. Comparison of force measurements across different molecules.

To assess the reproducibility of the measured switching force and interaction behavior, we present an additional example obtained on a different TBrPP-Co(II) molecule using an independently conditioned tip.

Because each experiment involves a distinct tip apex configuration, variations in the absolute magnitude of the measured total force $F_z^{total}(z)$ are expected. These variations affect both the tip-substrate and tip-molecule contributions, leading to differences in the raw force curves across datasets. However, after subtraction of the averaged tip–substrate interaction, the resulting tip-molecule force $F_z^{tip\text{-}moleculel}(z)$ exhibits consistent quantitative behavior across different

measurements. As shown in Figure S1, the background-subtracted force curves (green) display similar magnitudes and profiles compared to those reported in the main text. In particular, the depth of the force minimum (Figure S1, gray inset) and the corresponding potential energy well (Figure S2, blue inset) are comparable to the main-text example, confirming that the intrinsic tip-molecule interaction is reproducible despite variations in the total measured force. The threshold switching height differs slightly between datasets, which reflects differences in tip structure rather than changes in the intrinsic molecular response.

A notable difference in this example lies in the behavior of the force curves in the short-range regime. Here, the deviation of the measured force from the extrapolated power-law fit is less pronounced than in the main-text case. The measured force remains close to the fitted behavior up to the switching point, indicating that the deformation process is relatively weak and short-lived. In this regime, the measured force (green curve) remains above the extrapolated fit, in contrast to the main-text example where it falls below the fit. This distinction reflects different deformation pathways prior to switching. In the present case, the absence of significant upward deformation toward the tip suggests that the molecular framework does not undergo substantial elastic distortion. Instead, the pyrrolic unit transitions more directly, likely through a localized downward tilting motion. This leads to an effective increase in the tip-molecule separation and consequently a reduction in the attractive interaction prior to switching.

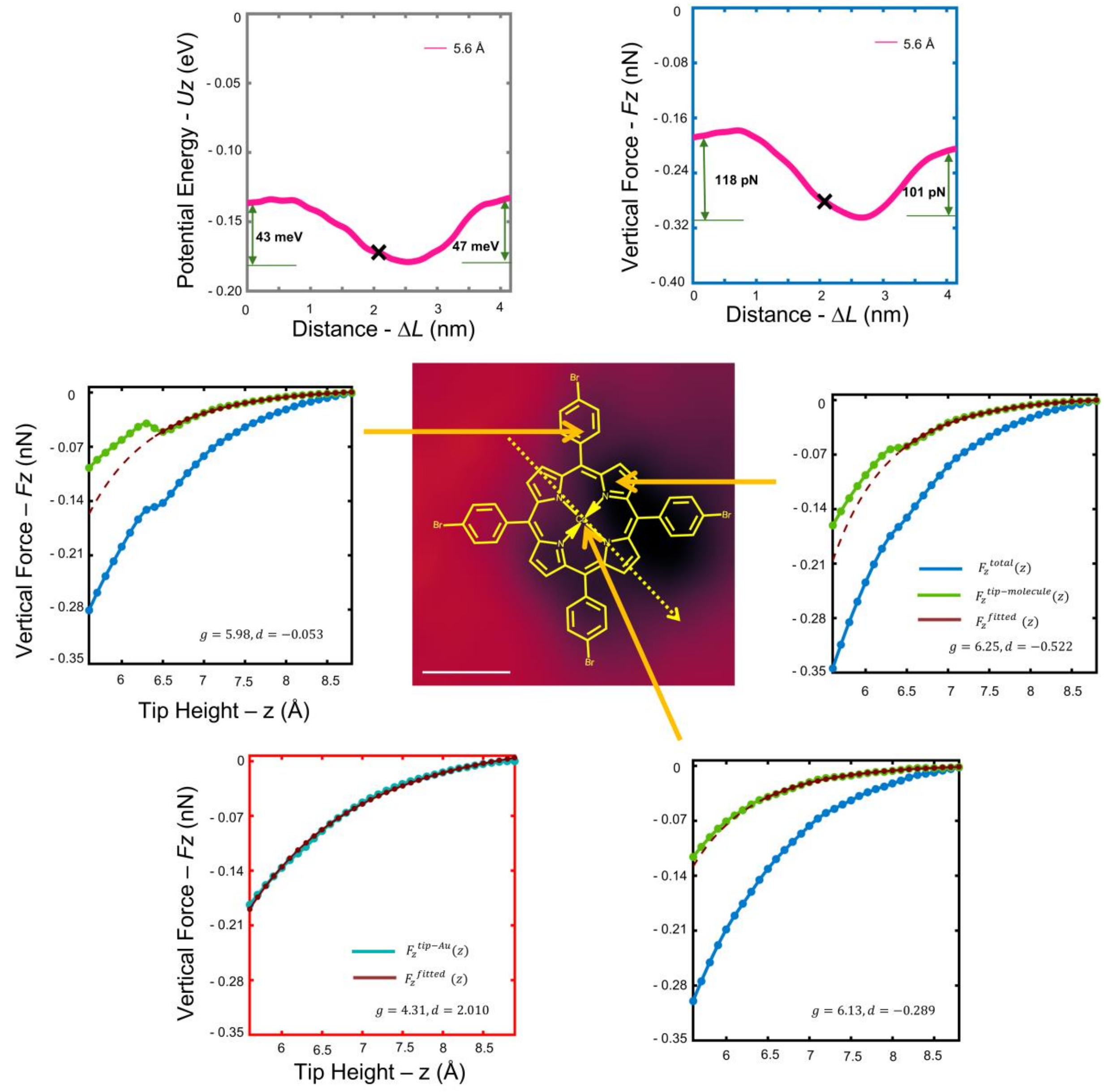

Figure S2. Insets highlight (blue) the depth of the force minimum and (gray) the corresponding potential energy well at the threshold height z = 5.6 Å, extracted from the line profile along the yellow dashed arrow, both of which are comparable to those observed in the main text. Isolation of tip-molecule force via subtraction of tip-substrate interaction, together with power-law fits (maroon) over the range z = 6.5 - 8.8 Å. Total force curves $F_z^{total}(z)$ (blue) were acquired at different lateral positions. The substrate contribution, obtained by averaging multiple curves on Au(111) (inset, red frame), is subtracted to yield the net tip-molecule interaction $F_z^{tip\text{-}molecule}(z)$ (green). Molecule structure is overlaid on the force map. Scale bar: 1 nm.

3. Inflection point test

The inflection point test evaluates whether the force deconvolution problem is well-posed or ill-posed in frequency-modulation AFM. The key criterion is based on the behavior of the force near its inflection point, where the curvature (second derivative) changes sign [65]. At such points, rapid variations in the force can lead to loss of information during oscillation, particularly if the oscillation amplitude is not appropriately chosen. The well-posedness of the reconstruction is quantified using the S-factor *S(F)*, defined as a function of the first and third derivatives of the force evaluated at the inflection point z_{inf},

$$S(F) = \frac{Z_{inf}{}^2}{4}\frac{F'''(Z_{inf})}{F'(Z_{inf})} \tag{S1}$$

A reconstruction is considered well-posed if the S-factor satisfies $S(F) \geq -1$. If this condition is not met, the validity of the reconstruction depends on whether the oscillation amplitude A lies within an allowable range determined by the local force variation, as described by:

$$A \leq L_{inf} \; or \; A \geq z_{inf}/2$$
$$L_{inf} = \sqrt{-F'(Z_{inf})/F'''(Z_{inf})} \tag{S2}$$

For this analysis, the vertical coordinate $z = 0$ corresponds to the closest approach point (threshold height) and thus does not represent the absolute tip-substrate distance used elsewhere in the manuscript.

As a representative example, we analyze the force curve acquired at the central position of the molecule (blue curve in Figure 4, top middle panel in the main text). The force data was processed using the publicly available implementation provided by Huber et al. [66], which automatically identifies inflection points z_{inf} and evaluates the corresponding S-factor. Two inflection points were

identified at z_{inf} = 0.256 Å and z_{inf} = 0.466 Å, corresponding to tip-substrate separations of 6.156 Å and 6.366 Å, respectively (Figure S3). Notably, these positions coincide with the onset of molecular deformation, where pronounced curvature changes are observed in the force profile. At z_{inf} = 0.256 Å, $S(F)$ = 0.43, which satisfies the condition $S(F) \geq -1$, indicating that the force reconstruction is well-posed at this point. At z_{inf} = 0.466 Å, $S(F)$ = −1.63, which violates this criterion. However, in this case, the oscillation amplitude used in experiment A = 50 pm falls within the permissible range $A \leq 18$ pm or $A \geq 23$ pm defined by the amplitude criterion in equation S2, ensuring that the reconstruction remains valid. This example illustrates both regimes of the inflection point test. Importantly, the same analysis was performed for all measured force curves, and in all cases the conditions for well-posed reconstruction were satisfied. These results confirm that the extracted force profiles are reliable and free from artifacts associated with ill-posed deconvolution.

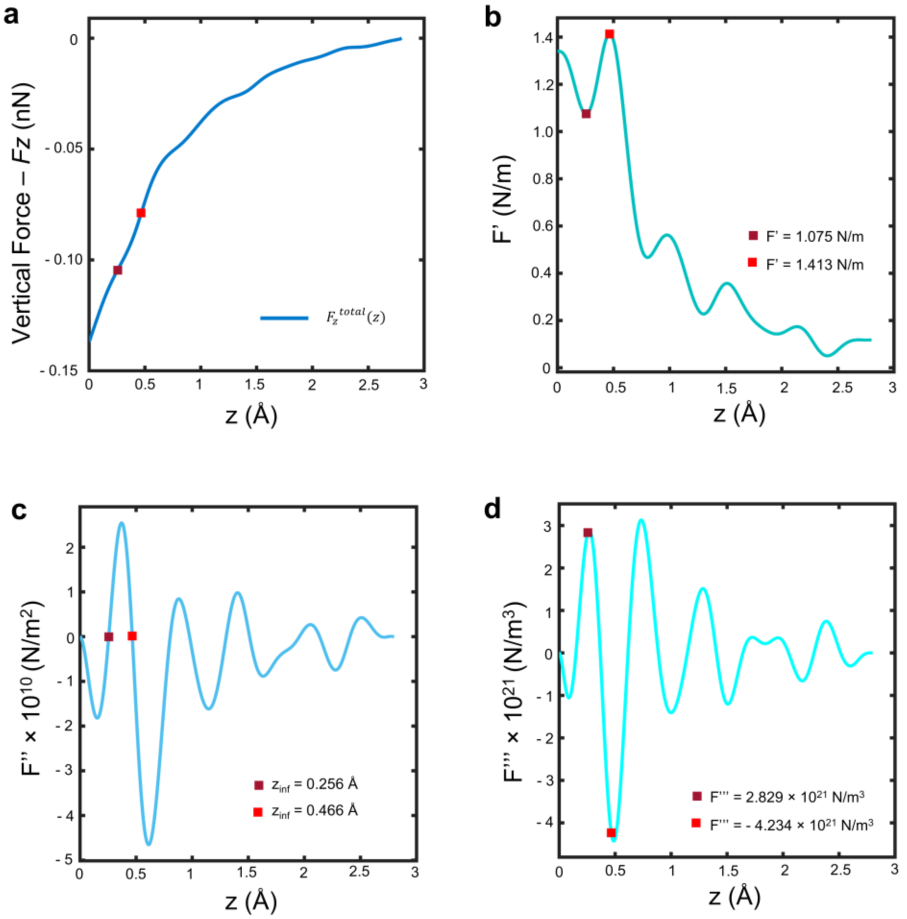

Figure S3. (a) Total vertical force curve $F_z^{total}(z)$ acquired at the central position of the molecule (blue curve in Figure 4, top middle panel in the main text). (b-d) First, second, and third derivatives of the force with respect to *z, F', F'' and F'''* as function of *z* plots, respectively.
